# High-Moisture Shear Processes: Molecular Changes of Wheat Gluten and Potential Plant-Based Proteins for Its Replacement

**DOI:** 10.3390/molecules27185855

**Published:** 2022-09-09

**Authors:** Nicola Gasparre, Marco van den Berg, Filip Oosterlinck, Arjen Sein

**Affiliations:** 1 Department of Food and Human Nutritional Sciences, University of Manitoba, Winnipeg, MB R3T 2N2, Canada; 2 Food Science Department, Institute of Agrochemistry and Food Technology (IATA-CSIC), C/Agustin Escardino, 7, 46980 Paterna, Spain; 3 Center for Food Innovation DSM Food & Beverage, Alexander Fleminglaan 1, 2613 AX Delft, The Netherlands

**Keywords:** extrusion, shear cell, plant protein

## Abstract

Nowadays, a growing offering of plant-based meat alternatives is available in the food market. Technologically, these products are produced through high-moisture shear technology. Process settings and material composition have a significant impact on the physicochemical characteristics of the final products. Throughout the process, the unfolded protein chains may be reduced, or associate in larger structures, creating rearrangement and cross-linking during the cooling stage. Generally, soy and pea proteins are the most used ingredients in plant-based meat analogues. Nevertheless, these proteins have shown poorer results with respect to the typical fibrousness and juiciness found in real meat. To address this limitation, wheat gluten is often incorporated into the formulations. This literature review highlights the key role of wheat gluten in creating products with higher anisotropy. The generation of new disulfide bonds after the addition of wheat gluten is critical to achieve the sought-after fibrous texture, whereas its incompatibility with the other protein phase present in the system is critical for the structuring process. However, allergenicity problems related to wheat gluten require alternatives, hence an evaluation of underutilized plant-based proteins has been carried out to identify those that potentially can imitate wheat gluten behavior during high-moisture shear processing.

## 1. Introduction

Meat has played a crucial role in Western diets, representing the principal source of high-quality proteins for humans. It still represents a base ingredient for many people across the world and with the global rise in prosperity, its consumption is expected to double by 2050 [1]. However, many studies have reported the negative effects on the environment, animal welfare and human health caused by modern meat production and its excessive consumption. Environmental problems such as waste, energy and water consumption, biodiversity loss, greenhouse gas emissions and climate change are imputable to current meat production [2]. Moreover, uncontrolled meat consumption could become an important problem for public health. Higher meat intake is associated with an increased risk of developing cardiovascular diseases, type 2 diabetes, and some types of cancer such as gastric and colorectal [3]. This has induced consumers to modify their dietary habits, limiting the intake of animal source products in favor of more plant-based foods. Diets richer in vegetables have been described as more environmentally friendly because of their lower carbon footprint compared with those prevalently composed of meat, especially beef [4].

Among the options proposed by the World Watch Institute to make the transition towards a more sustainable diet less difficult, meat analogues play a crucial role [5]. If consumers accept these new products as an alternative, recognizing their meat-like properties, meat analogues may become a powerful strategy for reducing meat consumption. Currently, the demand for meat substitutes is increasing and their global market is projected to grow at a compound annual growth rate of nearly 8% from 2021 to 2026 [6].

The term “meat analogues” refers to a large category of food products that try to mimic the texture, taste and appearance of animal whole-muscle meat and processed meat products such as burgers, patties, sausages, and nuggets [7]. Based on their source, meat analogues can be grouped into three main categories: cell-based (in vitro or cultured meat), fermentation-based (mycoproteins) and plant-based [8]. Alongside these principal groups, protein from microalgae (spirulina) [9] and insects [10] have started to be used in meat alternative formulations. Since ancient times, the presence of high-protein foodstuffs (tofu, tempeh, seitan) has been described in oriental diets; these products were obtained from soy and wheat protein via traditional processing methods [11]. In the sixties, with the advent of extrusion-cooking technology, protein concentrates from defatted soy and wheat gluten (WG) have been utilized for texturization at relatively low-moisture (20–40%). These products have a spongy-like appearance, should be rehydrated before eating and they represent the most widespread type of meat analogue in the market [12]. The soggy structure, in the form of granules or chunks, allows them to be mixed with other binding ingredients to prepare hamburgers and patties. Later on, extrusion with high-moisture content (40–80%) has been utilized to create products with a structure more closely resembling that of whole-muscle meat [13]. The extended cooling die helps fiber formation at temperatures lower than 75 °C, which results in a final product with a characteristic anisotropic fibrous structure close to that of animal meat [14]. Recently, many studies have been carried applying shear cell technology in the production of batch process meat alternatives with comparable attributes to those obtained via high-moisture extrusion [15,16]. Nowadays, the main proteinaceous ingredients utilized in meat-like products available in the market are provided from soy or pea, mushrooms and (WG), while extrusion-cooking represents the most employed technology [17]. The use of WG in high-moisture shear processes has allowed the obtention of meat analogues with fibrous texture and mouthfeel comparable to those of the whole animal muscle. Its well-known binding and dough forming ability, as well as its viscoelastic behavior appear to underlie the creation of an anisotropic structure when subjected to certain processing conditions [8]. Moisture content, temperature and shear have been identified as the main key drivers for the achievement of different structures with a wide range of fibrousness degrees [12]. In fact, the gluten contribution in achieving the desired textural features is strictly connected to the physicochemical interactions among gliadins, glutenins and other polymers present in the blend [7]. Health issues caused by gluten-related disorders underline the need to find a solution that can mimic the role WG plays within high-moisture shear processes. Among the possible WG substitution strategies, the use of an underutilized plant-based protein offers a valid alternative. Therefore, this review focuses on the recent scientific literature to clarify, at the molecular level, the role of WG in the structuring process of plant-based meat analogues obtained through high-moisture shear processes, such as wet extrusion and shear cell. To date, different studies have evaluated the effect on the textural quality of meat analogues after WG addition. Nevertheless, a comparative assessment between the different influence of wet extrusion and shear cell on WG fiber formation is still needed. The relevance of elucidating the uniqueness of WG in improving the textural features of meat analogues will be pivotal for the evaluation of the main underutilized plant-based proteins that could be used as potential WG substitutes. Moreover, the analysis of their structures and functionalities will be addressed, so as to identify new plant-based proteins for the production of meat analogues free from WG.

## 2. High-Moisture Shear Processes and Protein Conformational Changes

Plant protein functionalities depend on their three-dimensional conformation, primary structure, the extraction techniques, and their ability to form cross-links during extrusion. Usually, plant proteins are divided into three broad classifications based on their protein content, grits (typically for soy with 50% of protein content db.), protein concentrates (70% db.) and isolated proteins (90% db.). Due to their availability and low cost, their more effective gelation properties and more hydrophobic and hydrophilic amino acids that might mix with water or lipids to build the three-dimensional protein network, soy proteins are the most extensively used protein in high-moisture extrusion for meat analogue production. Lately, because of its stronger beany flavor and high allergenicity, soy protein has been partially replaced by pea protein with resulting products that were less allergenic with improved hardness, chewiness, viscoelastic characteristics, and fiber structure [18]. Both proteins are composed of globulin and albumin fractions and around 90% of the latter contains 7S globulins and 11S globulins [19,20]. Under adequate thermomechanical conditions, these two subunits are primarily responsible for the structural and functional changes of the protein in terms of solubility, gelation, emulsification, and foaming. Soy proteins are mainly composed of glycinin and conglycinin, while pea protein includes a combination of legumin, vicilin and convicilin [21]. In the case of soy protein, glycinin (11S) seems to be more important for the texturization process, while in pea protein, legumin (11S) is key [22,23]. The greater capacity of soy protein to become texturized, compared with that of pea protein, may be due to the higher content of the 11S fraction (glycinin), which is responsible for disulfide bond formation in the cooling stage. However, the only molecular reassociation of legume globular proteins is not sufficient alone to reach the fibrous texture and water binding capacity typical of the whole animal muscle [7]. Therefore, to overcome this problem, soy or pea protein are usually mixed with WG that, thanks to its elasticity and extensibility, makes the texture closer to that of meat-based products. WG still remains the most important protein-based texturing agent for fibrous structure creation because it promotes protein cross-linking and generates a three-dimensional macromolecular network via the disulfide exchange reaction [24,25]. In summary, the basic formulation used for high-moisture extrusion and shear cell technology includes soy or/and pea protein often combined with WG and water (40–80%).

Regarding the thermomechanical structuring methods employed for plant-based meat analogue production, the ones that are most employed bring together high temperature (95 to 160 °C) and shear blending. These techniques allow a protein matrix phase transition with the formation of an anisotropic structure with a fibrous nature, after cooling [26]. Extrusion and shear cell technologies are the most studied techniques capable of realizing a product with a meat-like texture. Furthermore, they share the main functional stages: mixing and hydration, thermomechanical stress, and cooling. The main difference between the two thermomechanical systems pointed out above is their design. Regarding extrusion, the proteinaceous matrix is transported along the extruder barrels due to the action of co-rotating screws. The barrels are under controlled temperature, and heating zones are provided in which the temperature increases from 25 to 160 °C, converting the mass into a state with melt-like properties when it heads to the die.

In the case of low-moisture (20–40% of water) extrusion, the melt that exits in the die experiences an immediate drop in pressure and temperature that causes an instantaneous water flash off. This results in an expansion of the products with a consequent formation of the distinctive puffed-spongy structure. Concerning the high-moisture extrusion (40–80% water), a cooling die is positioned at the exit of the extruder barrel, to limit the expansion of subcritical water in extruded materials. During this crucial step, the decreasing of the temperature and viscosity allows the biopolymeric material to be shaped inside the die channel. On the other hand, the shear cell process is conducted by a shearing device, based on the layout of the rheometers, in which high shear can be produced in a cone-in-cone or in a concentric cylindrical arrangement [27].

The physicochemical transformations that occur during these thermomechanical treatments are strongly dependent on the process parameters and composition. Protein matrix characteristics (pH, ionic salt concentration), temperature, shear rate, water content and the presence of fat and polysaccharides impact the rheology of the proteinaceous melt [28]. The viscosity of the melt is a result of different interactions between protein-protein, protein-water, protein-polysaccharide, and protein-lipid that contribute to the texture and sensorial attributes of the final products [29]. During the thermomechanical processes, the material goes through different rheological states, in particular the proteins undergo conformational changes that modify their native arrangement. Here, the unfolded molecular chains exhibit their hydrophobic amino acids that were initially surrounded by the molecules [30]. As the temperature increases, the flow rate of the melts reduces, owing to newly formed protein-protein and protein-water interactions that promote viscosity increments [24,31]. This step is crucial for the quality characteristics of the final products; in fact, in addition to the association and/or aggregation, degradation of the protein molecular chains may also occur [32]. The temperature reached during the cooling step (below 75 °C) helps the rearrangement and cross-linking of the protein molecules assuring the melt laminar flow [33]. Some studies about the extrusion process demonstrated that the interactions that sustain the original configuration of the protein were changed by the thermomechanical energy, but the major chemical links, such as peptide bonds, did not undergo any change [23,34]. When soybean protein isolate was extruded with a different moisture content (28 and 60%), hydrophobic interactions, hydrogen bonds and disulfide bonds were the main links that held the structure of the extrudates [35].

Whereas the essential processing stages of the main high-moisture shear processes were found to be rather similar, the most utilized raw materials for meat analogue production (i.e., soy and pea proteins) are not sufficient to guarantee a fibrousness match to that of animal meat.

## 3. Main Process Parameters Related to Protein Conformational Changes

### 3.1. Impact of Temperature on the Protein Molecular State

Among the process parameters that most affect protein structure, temperature plays a key role [31]. When the temperature gradually increases, initially the hydrogen bonds break down and water molecules penetrate, allowing the protein chains to gradually unfold [34]. When the heating sharply rises, a disruption of the intramolecular disulfide bonds primarily occurs followed by the formation of new intermolecular disulfide bonds. When the temperature is above 150 °C, these new disulfide bonds are then disrupted, thus increasing the number of free thiol groups [14]. The barrel temperature is responsible for ensuring the complete melting of the material and the subsequent fiber structure formation. In high-moisture extrusion and shear cell processing, to guarantee the passage of the melt through the cooling die and for successful fiber formation, the temperature should be kept below 75 °C [23]. Indeed, gradual cooling in the cooling zone affects the flow velocity during solidification and represents a crucial point for the success of the final product [36]. This guarantees that the melt is in a laminar state, in which its temperature and flow velocity are higher at the core of the flow channel than those of the zones closer to the cooled wall [23]. Under these conditions, the melt exhibits a multilayered arrangement with layers parallel to the cooling die [32]. For high-moisture texturized soy protein, the fibrous structure can only be created when the melting temperature is above 130 °C [32]. At a melting temperature below 120 °C, the structure of high-moisture texturized soy protein was easier to break, and the shape was not uniform [37]. As reported by Zhang, et al. [38], an increase in the melting temperature from 130 to 150 °C, provoked a rise of the degree of texturization, which suggested that the material became fully melted, progressively improving the protein-protein and protein-water interactions. When the temperature was increased from 150 to 160 °C, the degree of texturization diminished and the surface of the samples presented small pits and a brownish color. These findings indicated that a relatively higher temperature may lead to a breakdown of the intermolecular bonds with a consequent degradation of the protein [14]. When the temperature was above 160 °C, the melt that resulted was hard to shape. At temperatures between 140 and 160 °C, the tensile strength of the extruded material was affected, while no effects were observed regarding the hardness and chewiness [39].

The secondary structure (*α*-helix, *β*-pleated sheet, or random coil) can be affected during thermomechanical processes, but few studies have analyzed the effect of extrusion on the secondary structure of proteins. To better understand the secondary structural alterations of soybean protein during extrusion, the Fourier-transform infrared spectroscopy method was adopted [40]. Outcomes indicated that during the heating process (between 120 and 160 °C), the *a*-helix was the most unstable structure and when the temperature reached 140 °C, it turned into a more stable structure. Nevertheless, the *β*-sheet persisted basically unchanged until the barrel temperature increased above 140 °C and the sub-stable β-sheet started to transform into a random coil, while the β-turn kept their structures unchanged even at 160 °C. Not unimportantly, most of the protein (especially isolates) used for meat analogue production comes from wet fractionation processes. This often has a negative impact on protein functionality due to the methods involved, such as alkaline or acidic extraction, iso-electric precipitation, ultrafiltration, and spray drying). Dry fractionation methods offer a suitable alternative; although it produces less pure protein fractions, it allows for the retention of native functionalities [41].

### 3.2. Water as a Plasticizer

The importance of the water function within thermomechanical processes has been described in the literature. Owing to its action of reducing glass transition temperature with the consequent increase of the molecular mobility of the protein, the role of water as a plasticizer shows it to be of critical importance for the physicochemical transformation of the proteinaceous matrix [42]. In addition to determining the melt viscosity, water is pivotal for the friction and operates as a thermal and mechanical energy transfer medium [32]. Adjusting the water content, based on the raw material requirements, is a valuable aspect to consider. Although the raw materials used for meat analogue production typically have a moisture content below 10%, adequate water amounts introduced to the multiphase system allows for the full use of the water functions. On the molecular scale, water molecules penetrate the protein matrix and hydrate the biopolymer chains (proteins and soluble carbohydrates). Interacting via hydrogen bonds with the easily accessible polar amino acid side chains and carbohydrates, water breaks down the protein-protein and carbohydrate-carbohydrate interactions, thus inducing a plasticization effect [43]. Then, under the prevailing temperature, the shear force and pressure conditions lead the transition to the melt-like state.

A degree of texturization growth of soy protein was observed when the moisture content increased from 35 to 50% [44]. Chen, Wei and Zhang [35] reported the changes of chemical cross-linking and molecular aggregation of soybean protein isolate during low (28%) and high-moisture (60%) extrusion. The authors reported that increasing the water content may increase the interactions between disulfide bonds and hydrogen bonds, as well as between disulfide bonds and hydrophobic interactions. At a moisture content of about 55%, the peptide chains become more easily stretchable and align better, because of the mobility enhancement of the key structure of the protein. Under high temperature conditions, water molecules promoted the transition of *α*-helix to *β*-turn, and *β*-sheet to random coil, and served to reduce the temperature necessary to form this network. The polymerization reaction, caused by a rise in the reaction rates of proteins, could be sped up by increasing the water content in the range of 20–40% [42].

In summary, water performs as a dispersion medium, plasticizer, and solvent, influencing the aggregation of the polymers and the melt viscosity.

### 3.3. Impact of Shear Force on the Protein Molecular Interactions

Another parameter that impacts structure formation in the thermomechanical process is the shear force occurring in the extruder barrel and at the extruder exit. It is reliant on the mechanical energy supplied by the extruder motor, and it is mostly used by the material in the filled parts of the extruder to dissipate heat through friction and viscous flow. The amount of mechanical energy applied to the ingredient mix is measured in terms of Specific Mechanical Energy (SME). The first impact of SME concerns melt viscosity: a higher level of SME leads to a lower melt viscosity and a higher melt temperature. As mechanical shear increased, the resulting higher SME was found to enhance the severity of the extrusion, thereby reducing the size of the macromolecules and inducing an increase in protein solubility in the phosphate buffer [45]. Vaz and Arêas [46] reported that shear force can break up the disulfide bridges leading to the exposure of the thiol groups attached to amino acids, such as cysteine. These thiol groups can be oxidized to form new disulfide bonds, thus promoting the formation of large protein aggregates [46]. Applying a lower shear rate is believed to lead to undeveloped polymerization because the collision rate of the protein molecules is reduced with fewer disulfide bond rearrangements. On the other hand, a higher shear rate has often been associated with a fragmentation of the matrix [45]. In combination with a weak shear force, the main interactions between proteins in texturized soy protein were disulfide bonds and non-covalent interactions; however, covalent cross-linking may occur when strong shear force is applied [42]. The time in which shear stress was applied contributed to the structure formation, having a positive impact on the yield stress of the matrix [47]. Using a capillary rheometer, Beck, et al. [48] evaluated the effect of heating and shear force (130 °C, 20 min and 3000 s^−1^) on pea protein isolate. The authors reported that larger peptides were broken when higher shear rate and temperature were applied, obtaining a flexible structure similar to that produced via high-moisture extrusion from the same ingredient.

Therefore, high moisture shear processes are thought to involve a balanced interaction between temperature-induced polymerization and shear-induced depolymerization. Denaturation, dissociation, unwinding, and the alignment of polymer chains all contribute to the production of a homogenous protein melt during the process. The amino-acid sequence and the amount of plasticizer employed all influence the covalent cross-links, which are fundamental for the structure formation.

## 4. Understanding the Fiber Formation Process

**Figure 1:**
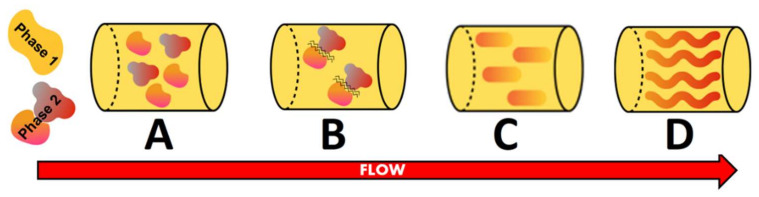
Structuring process steps adapted from Dekkers, et al. [49]. (**A**) = phase separated; (**B**) = breaking-up of the film; (**C**) = formation of merged droplets; (**D**) = alignment.

Fiber formation during thermomechanical processes could be better understood through analyzing the structuring processes. After blending, water migrates between the two phases, leading to a change in both volume fraction and consistency of the phases (Figure 1A). This could happen in a simplified conceptual picture where, for instance, only the proteinaceous ingredient may hold, but as soon as it is mixed with some other raw material (WG), there are at least three phases: the protein, the carbohydrates (residues of the proteinaceous raw material) and the WG, which is thermodynamically different from the other protein present in the formulation. The parameters that affect this water redistribution are temperature, process history and the presence of other components (salts or sugars). Most of all, the nature of the biopolymers and their affinity for water are determinant [49]. Seen as a water-in-water emulsion, the low interfacial tension between the phases makes them deform easily [50] and hence can be neglected as a factor in the process of fiber formation. Deformability and breaking of the dispersed domains directly depend on their size, phase viscosity and shear rate. To deform the domains, the viscosity ratio between both phases needs to be balanced. Then, two domains start to approach each other with the consequent film drainage and breaking up of the film, which leads to a formation of a merged domain (Figure 1B,C) [49]. Owing to the shear flow, the particles and fibers are aligned, resulting in a change from a structural standpoint (Figure 1D) [51]. The interaction of the fibers among each other, the aspect ratio of the fiber (length over width) and the type of flow during the process represent the main variables that influence fiber orientation [49].

### Two Models: Phase Separation in Barrel and Die Head versus Phase Separation in Cooling Die

**Figure 2:**
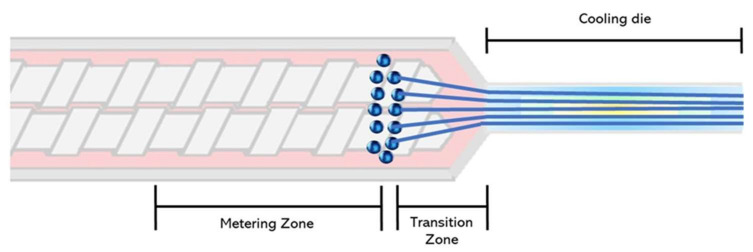
Fiber formation model adapted from Mitchell and Areas [52], with phase separation occurring in the extruder barrel.

**Figure 3:**
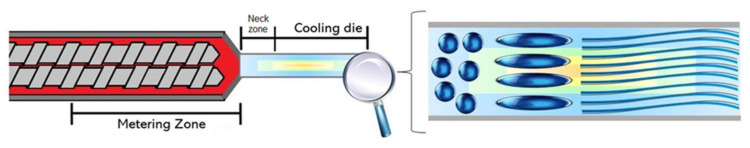
Fiber formation model adapted from Sandoval Murillo, et al. [53], with phase separation occurring in the cooling die.

To mechanistically explain the fiber formation process in the extruder, Mitchell and Areas [52] proposed the suspension model. According to this model, the biopolymer melt forms two highly concentrated phases: a homogenous continuous phase and a dispersed phase. The authors explained that the phase separation occurs at the extruder metering zone (Figure 2). Later on, Sandoval Murillo, et al. [53] proposed another model in which the phase separation that gives rise to the layered structure, takes place in the cooling die and is influenced by the temperature gradient (Figure 3). The authors reported that the temperature of the melt is super-critical in the extruder barrel and no phase separation is observed; once the melt enters into the cooling die, the reduction in the cross-section causes a flow speed increase. Here, the material is cooled down and its thermal diffusivity is the same as the water while the temperature becomes sub-critical, producing a phase separation. This phenomenon starts from the zones closer to the extruder walls producing a structure oriented towards the extrusion direction [53].

## 5. Role of Wheat Gluten in Plant-Based Meat Analogues Obtained via High-Moisture Shear Processes

### 5.1. Mechanical Properties of Wheat Gluten

Since meat analogue formulations are mainly composed of plant sources rich in protein (50 to 95% db.), the physicochemical transformations in the protein phase that occur during thermomechanical processes are responsible for the fiber formation, while other ingredients utilized in commercial formulations (polysaccharides, lipid, salts, or solvents) can help the structuring process [28,54]. In particular, polysaccharides also contribute to phase separation and promote competition for water, while lipids are responsible for the torque reduction, which causes unwanted slip and uneven flow in the cooling die. As reported by Beniwal, et al. [26], in the twenty-eight commercial samples of meat analogues that were reviewed, fifteen included WG in their formulations. In wheat, the largest portion (≈80%) of the total protein content is represented by the WG; its equivalent form is also present in other grains such as barley and rye [55]. WG development occurs when the glutenins and gliadins are mixed with water and exposed to mechanical stress [56]. Because of its impact on the technological properties of wheat in the bakery field, these proteins have been the object of several studies during the last decades. Nevertheless, WG also produces adverse effects on human health related with allergies and intolerances. From a technological standpoint, it is commonly accepted that glutenin is responsible for the polymeric network formation, while gliadin acts as a plasticizer contributing to the viscosity and extensibility of the dough system [57]. Glutenins, which are insoluble in aqueous alcohols, are characterized by a high molecular weight with extended structures that become alcohol soluble only when the disulfide cross-links are broken through the action of some reducing agents [58]. Depending on their molecular weight, glutenins are divided into high and low-molecular-weight glutenin subunits [59]. Via intermolecular disulfide bonds and non-covalent interactions with gliadins (hydrogen bonds, hydrophobic interactions, and ionic bonds), the high molecular weight subunits build macropolymers, that provides strength to the structure [60]. Gliadins, that are alcohol soluble and have a relatively smaller molecular weight, are subdivided into α-, γ- and ω-gliadins in accordance with their different electrophoretic mobility [61]. In normal conditions, owing to their cysteine residues, the α- and γ-gliadins principally connect via intramolecular disulfide bonds [62]. The resultant viscoelastic properties make WG also suitable for the improvement of meat analogue products obtained through extrusion. In fact, in the presence of water during kneading, a continuous cohesive phase is formed [63]. The existence of the new WG rubbery bands will separate from the other phases causing their folding in an elongated network oriented toward the extrusion flow direction. Other authors have pointed out the importance of WG in creating a strong and elastic gel, which would bind the fibers together guaranteeing the final product’s strength; for this reason, it is employed in plant-based meat analogue formulations as a thickener, fortifier, and texturizing agent [17,64].

### 5.2. Wheat Gluten Behavior in High-Moisture Shear Processes

As WG is still one of the most used co-products blended into soy-based wet extrudates, a deeper elucidation of its behavior during thermomechanical processes is needed. A scientific literature review spawned heighten studies (Table 1) about WG performance when subjected to closed cavity rheometer, shear cell and high-moisture extrusion. The selected scientific articles focused on WG in combination with soy protein isolate and concentrate (SPI and SPC), pea protein isolate (PPI), mung bean protein isolate (MBPI), peanut protein isolate (PNPI) and rapeseed protein concentrate (RPC).

In order to simulate extrusion-like conditions, Emin, et al. [42] utilized a closed-cavity rheometer, investigating the process parameters involved in reactivity changes of the reaction behavior of a highly concentrated WG model system. The results showed that temperature, water content, shear and a step change in shear produced the main changes in WG reaction behavior. Changes in the complex modulus results illustrated the presence of three characteristic regions for all the tested samples, depending on the temperatures applied. At mild temperature conditions, a decrease in the complex modulus was observed mainly due to higher molecular mobility. Subsequently, a sharp increase was noticed when the temperature and shear force were increased. This stage is related with the polymerization reaction, which involves first glutenins and after gliadins that form a cross-linked network [79]. Samples with 40, 30 and 20% of water content achieved the highest value of the complex modulus (peak) at about 130, 135 and 140 °C, respectively. At these conditions, the structure was fully formed. The last stage, where the temperatures were even higher, a final decrease of the complex modulus was observed, which corresponded to the end of the polymerization reaction, or to the onset of a degradation reaction [80]. The evaluation of the sodium dodecyl sulphate (SDS) extractable protein, under non-reducing and reducing conditions, indicated that the WG polymerization reaction was based mostly on disulfide bond rearrangement and was not influenced by the application of different shear rates (up to 50 s^−1^). Moreover, a decrease in SDS extractable protein under non-reducing conditions was observed when increasing the treatment time and temperature were applied [65]. This decrease was principally ascribed to the formation of new gliadin-glutenin cross-linking [81]. When Pietsch, et al. [24] explored the influence of processing conditions (temperature and pressure) on changes in WG polymerization during high-moisture extrusion, they found that the main modifications occurred in the screw section and not at the die. When the extruder pressure was varied from 1.5 to 3.5 MPa and specific mechanical energy from 32 to 206 kJ kg^−1^, no impact was observed on the WG polymerization reaction. On the other hand, when the temperature was varied between 90 to 160 °C, changes in polymerization behavior were observed, also visible in the appearance of the final products: samples treated at 110 °C showed a certain grade of isotropy, while at a temperature of 145 °C, anisotropy prevailed. During the high-moisture extrusion process, anisotropic structure development is the function of WG polymerization. Samples with a more marked anisotropy, hardness and Young’s modulus were obtained at higher polymerization levels. Moreover, the microscopic evaluation confirmed that the formation of the anisotropic structures was due to the presence of a dispersed phase [72].

Jia, et al. [77] reported the results of the SDS-PAGE analysis of WG subjected to high-moisture extrusion when adopting different parameters (Table 1). The amount of the high-molecular-weight glutenin subunits increased while the amount of their lower molecular weight counterparts decreased, as well as the number of free sulfhydryl groups. Nevertheless, the molecular weight of the glutenin subunits decreased as the screw speed and flow rates increased. In particular, when the barrel temperature increased, the *β*-sheet structures of WG increased, while the *α*-helices and *β*-turns oscillated with the consequent formation of a tighter WG network. Confocal laser scanning microscopy underlined that the samples processed at higher temperature had a denser WG network, which may be caused by the polymerization of low-molecular-weight glutenin subunits.

To improve the strength of the WG network, Chen, et al. [82] blended it with peanut oil (2%) before undergoing low-moisture (25%) extrusion. Results from the scanning electron microscopy showed that the samples with this (low) level of peanut oil had a higher particle size than those of simple WG. In fact, during thermomechanical processes, heating causes a change of the initial protein structure favoring a higher exposure of the hydrophobic portions. Due to this, more lipophilic sites are available for the interactions between the hydrophobic branched chain of protein and the hydrophobic group of lipids that lead to WG aggregation improvement [83]. Based on these observations, one might speculate that low levels of lipids may act as a sort of plasticizer, too.

### 5.3. Biopolymer Incompatibility Helps

As pointed out above, many of the wet-extruded products consist of soy-based raw material and seem to profit from the presence of WG; for this reason, they are considered the current standard in meat alternative formulation. Under normal atmospheric conditions, soy proteins are characterized by a strong gelation property that, during thermomechanical processes, may form firm networks, due to some dissociation and reassociation mechanisms [84,85]. Furthermore, the improvement of the three-dimensional protein network development with water or some lipids (2%) is favored by the presence of hydrophobic and hydrophilic amino acids [86].

Moreover, Grabowska, et al. [67] reported that through a shear cell device, using soy protein isolate (SPI) (30%), a brittle and porous gel was formed in the absence of shear, while when shearing was applied, a firm and dense gel was formed. In the case of WG presence (30%), randomly oriented small fibers were observed in the absence of shear, but when mechanical energy was applied, a clearly fibrous shear direction-oriented structure was noticed. Blending SPI with WG (both at 15%) without any shearing led to a gel formation with short thin fibers; on the contrary, gel with long, thick, and thin fibers in the shear direction were reported when shearing was applied. Anisotropy was reported in the samples with a protein concentration of 25 and 30% where the SPI-WG ratios were 4:1 and 3:2. The authors concluded that the formation of two separate phases of incompatible biopolymers (SPI and WG) is crucial for structure development, and in addition, shear flow provided a deformation and alignment of the phases, generating the typical layered structure of the products from a wet process under shear (shear cell or extrusion). Similar phases rheological properties are essential to deform and align the dispersed phase to obtain the anisotropic structure. For example, in a multiphase system (SPI-WG) subjected to a shear structuring, SPI absorbed more water than WG, making the volume of the SPI phase larger than the mass fraction; consequently, both phases reached similar rheological characteristics [69]. Each formulation appears to have an optimal time, shear, and temperature domain. When SPI-WG were blended at a ratio of 3.3:1 at high-moisture content (69%), the process time and rotation rate turned out to be not critical. Only temperature affected fiber formation; fibrousness was evident at temperatures between 90 and 100 °C [68]. Once the temperature was kept constant at 120 °C, the duration and rotation rate of the applied flow regime appeared crucial for structure formation. The best results in terms of fibrous structure were achieved with a thermomechanical process of 30 min and a rotation rate of 20 rpm. Below and above these optimal conditions, the samples were not adequately structured or deformed, respectively [15]. Schreuders, et al. [70] compared the structuring potential of SPI and pea protein isolate (PPI) blended with WG (50:50) with high-moisture content (60%) varying the process temperature (from 95 to 140 °C) and leaving the shear rate and process time unchanged. No fiber formation was observed at temperatures below 120 °C for the PPI-WG blends. Regarding the SPI-WG samples, anisotropic character was developed in the temperature range of 110–140 °C. Mechanically speaking, the resultant blends with SPI were three times stronger than the PPI blends at 120 °C, because of the different capacity to form a strong phase, which was responsible for the deformation and arrangement of the protein in the flow direction. Moreover, the low viscosity of the PPI-WG phase seemed to be less able to retain the air bubbles compared to the SPI-WG system. At 140 °C, this strength difference was equalized. At 130 °C, a strength decrease was observed in both samples and the PPI-WG treated at 110 to 130 °C obtained a similar chicken meat tensile strength. The best results were obtained when there was a balance between right fluidity, phase separation and shear alignment. The authors reported that formulations containing PPI and WG can represent a valid alternative to produce fibrous products with texture attributes similar to those of cooked chicken meat [70]. A similar trend was reported when WG was used in combination with RPC and processed through a shear cell. Compared with SPC, the combination of RPC with WG offered a wider range of fibrousness in the final products [71].

To reproduce the thermomechanical conditions occurring during and after extrusion, a closed-cavity rheometer was employed. Through the plasticity of the model, the matrices composed of SPI, PPI and WG was studied [66]. The authors reported that the modulus of WG increased during heating and after cooling it remained elevated. A completely different pattern was observed in SPI and PPI, where the moduli decreased during heating. The analysis of the energy dissipation ratios related with the plasticity of the materials showed that upon heating, PPI lost its elasticity earlier than SPI, while WG exhibited sharp dissipation after broad deformation.

These outcomes agreed with the behaviors observed during flow-induced structuring, in which SPI and PPI produced a homogeneous matrix while WG formed stretched fibers. During extrusion, the elasticity appeared to be influenced by the moisture content; when this was increased (from 40 to 55%) the elasticity decreased [25]. Chiang, et al. [73] observed that changing the ratio of soy protein concentrate (SPC) and WG produced structural changes in texture, fiber structure, hardness and chewiness of plant-based meat analogues processed via high-moisture extrusion. Because of their higher texturization degree, samples containing the highest WG concentration (30%) had texture remarkably close to that found in boiled chicken breast. The authors also found that the disulfide bonds increased when the WG content was higher, thus improving the structure strength. The same trend was confirmed by Samard, et al. [74], who reported that WG incorporation, screw speed and water content produced changes in the physicochemical properties of the SPI-based samples. Screw speed affected only the springiness, while when low-moisture extrusion was adopted without WG, the final products presented an expanded structure with the lowest texture stability. On the other hand, the WG inclusion helped the formation of a spongy structure. Only when high-moisture extrusion and WG incorporation were implemented was the resultant structure of the plant-based meat analogue denser, with a more stable texture and with a higher degree of fibrousness. The tighter structure developed by WG in high-moisture extrusion improved the retention of flavor and water [76]. Intermediate moisture (50%) extrusion was applied to texturize different proteins: SPI, mung bean protein isolate (MBPI), peanut protein isolate (PNPI), pea protein isolate (PPI) and WG. SPI and WG-based samples showed better textural properties, and MBPI and PNPI-based samples were lower in rehydration and textural characteristics, while PPI formed a sponge-like structure with attractive properties in terms of rehydration, emulsifying capacity, oil absorption and texture [75].

In conclusion, next to its already known viscosity and elasticity properties, WG assumes significant importance in controlling the characteristics of the final products obtained through high-moisture shear processes. Its polymerization is able to generate different degrees of anisotropy depending on the main process variables, such as temperature, hydration and applied shear. Although the most used protein (soy and pea) in plant-based meat analogue production merely form products with a more brittle texture, WG contributes to the improvement and strength of the structure through the creation of new disulfide bonds, thanks to the presence of a higher number of cysteine residues. Disulfide bridges can be intramolecular, mainly when gliadins are involved, though intermolecular bridges with glutenins are also implicated. So, at this point it seems logical that the ratio between gliadin and glutenin plays a pivotal role in determining WG’s final functionality. Another fundamental characteristic that WG brings in a multi-phase system is its thermodynamic incompatibility with the plant-based protein, which is essential for the structuring process.

## 6. Under-Explored Plant-Based Proteins as a Potential Wheat Gluten Substitute in Meat Analogues

The food industry and academia are both interested in managing and processing food wastes and by-products. So, mainly treating residues for starch and oil production to recuperate desirable components, such as protein, represents a valuable strategy towards “zero waste” production. Moreover, there is a drive for recuperating potential allergens in food production. In the following section, the principal outcomes from under-utilized plant-based protein, especially those from by-product matrices will be addressed, as well as their potentiality as WG replacers for plant-based meat analogue formulations.

### 6.1. Zein and Similar Proteins

Zein, from maize, has received much attention because it shares many features with WG. Indeed, zein is a water-insoluble prolamin that can self-build a viscoelastic matrix when hydrated and heated above its glass-transition temperature [87]. Once this critical temperature is reached, zein may be pulled, stretched, and shaped, representing a potential opportunity to produce different valuable structures for plant-based meat analogue applications [88]. Available as a by-product of corn starch and corn syrup production, zein consists of *α, β, γ* and *δ* fractions with a great amount of non-polar amino acids [89]. As described in the above section, WG network formation is primarily dependent on the disulfide bonds formed between the cysteine residues of glutenins and gliadins, while hydrogen bonds and hydrophobic interactions improve the strength [90,91]. In the case of zein, the network formation is mainly performed by non-covalent interactions [92]. Because commercial zein contains mostly *α*-zein, which is characterized by only one or two cysteine residues per subunit, the difference with WG, in terms of structure and rheological properties, is even more striking [93]. Mattice and Marangoni [88] studied the self-assembled zein networks from a fundamental structural and rheological standpoint. Zein networks were examined independently of complex food matrices. Results showed that zein needed at least 24 h to fully form the network and intramolecular *β*-sheets predominated during the early stage. After the addition of water, zein rapidly aggregated in a large size, in order to reduce the surface regions exposed to the aqueous system. As time passed, the formation of non-covalent hydrophobic interactions strengthened the network. Interestingly, when zein was subjected to heating (60 °C) in an excess of water, it mechanically elongated and added (1.5 and 3%) to a model system of tofu-like SPI gel. Overall, the outcomes proved that SPI gel containing 3% of mechanical elongated zein had similar texture characteristics to chicken meat, and fibers with a uniform diameter from 1.5 to 2 μm and an orientation that most contributed to the meat-like texture [94]. Zein has also been successfully used in plant-based cheese formulations, in particular, an incorporation level of 30% produced samples that displayed similar performances to cheddar cheese regarding texture, rheology and in melt-stretch qualities [95]. Similar changes found in the WG viscoelastic matrix were observed when zein was hydrated above its glass transition temperature (35 °C); in fact, a viscoelastic structure, based on β-sheet arrangement took over from its native organization (α-helices). Nevertheless, when zein and WG were subjected to shear stress, they behaved differently. After the applied stress, WG kept the viscoelasticity longer, whereas zein lost it quickly; this difference depended on the higher content of β-sheet structures found in WG. In the future, this limit could possibly be overcome by coupling zein with another protein capable of stabilizing the system through the formation of β-sheets [96]. The ability of zein to form elastic networks under heating conditions and its capacity to induce phase separation enrich this protein with new functionalities to be exploited for the production of plant-based meat analogues. Nevertheless, additional research is needed to better understand the impact of zein on the texture of the final extrudates.

Other prolamin-rich protein fractions include kafirin from sorghum, which seems to have higher glass transition temperature than zein or WG [97]. In the study carried out by Xiao, et al. [98], small angle X-ray scattering, and atomic force microscopy showed that kafirin aggregated through non-covalent interactions into elongated, ellipsoidal structures, even in good solvents. Elhassan, et al. [99] reported that when kafirin was dissolved in glacial acetic acid and the protein was precipitated by fast coacervation with cool water under low shear, stable viscoelastic masses were created. According to the authors, the existence or reduced expression of the cysteine-rich and -kafirin subclass in the kafirin influenced dough appearance but did not affect the stress-relaxation behavior. For this reason, the degree of polymerization of kafirin is not crucial for its ability to form a viscoelastic mass.

### 6.2. Leaf Proteins

Recently, leaf proteins are attracting a certain interest especially from a nutritional standpoint. These proteins can be extracted from various crops, green by-products, and aquatic plants. The soluble portion is principally constituted from ribulose-1,5-bisphosphate carboxylase/oxygenase (RuBisCo), which is an enzyme that provides carbon fixation [100]. This oligomer contains more sulfur amino acids than wheat proteins; moreover, it is rich in lysine, threonine, and tryptophan [101]. In addition to its nutritional benefits, RuBisCo is able to form gels at low concentration and low temperature in aqueous environments and it is recognized for its relatively good solubility. Covalent bonds (disulfide bridges) can develop during the heating phase, but noncovalent interactions such as hydrophobic interactions and hydrogen bonds are pivotal during the cooling phase. When RuBisCO was assessed via small deformation rheology tests, its G′ increase was substantially higher than that observed for the other proteins (whey and egg) [102]. Using a dynamic thermomechanical analysis, the impact of RuBisCo on the mechanical properties of wheat-based dough was investigated and compared with those that included WG and PPC [101]. RuBisCo behaved differently in comparison with WG and PPC. Owing to its reactivity and lower competition with starch for water absorption, RuBisCo maintained the dough elasticity during heating as opposed to WG and PPC. The high concentration of free thiol groups in RuBisCo helped to form sulfhydryl-disulfide interactions with WG building a co-protein network. Despite its great potential and its ease of finding, RuBisCo still represents an underutilized protein source. Its higher sulfhydryl group content and its superior gelling properties could make RuBisCo an interesting alternative to WG in plant-based meat analogue formulation production; nevertheless, further evaluation of the extraction process is needed, since it strongly affects the final functionalities of the protein. The heat-induced aggregation ability with consequent network formation allows RuBisCO to generate gels with distinct properties that under severe thermomechanical conditions may promote phase separation, contributing to fiber formation. More research regarding the improvement of protein extraction techniques could help in reducing the cost and in obtaining more purified products.

### 6.3. Oilseed Proteins

Cruciferin and napin, the two major storage proteins contained in rapeseed, have demonstrated excellent foamability, emulsion-stabilizing and gelation properties (specifically high molecular weight cruciferin) [103]. Cruciferin is composed of a hexameric quaternary structure made of two trimers composed of cruciferin subunits that are disulfide-bridged. Each subunit of pro-cruciferin (11S globulin) from Brassica napus (3KGL) contains five cysteine residues; four cysteines form two disulfide bonds leaving one free cysteine. On the other hand, napin (2S albumin) consists of two subunits linked by two disulfide bonds with no free cysteine available [104]. Recently, rapeseed proteins have demonstrated their aptitude to form weak gels when treated with high pressure or heating [105]. Heating temperature and pH have been identified as a key factor able to affect the gelling properties. Perera, et al. [106] reported a hydrophobicity increase in the cruciferin when treated at low pH, while under alkaline conditions, it showed a certain thermal stability that was lost when the pH was around 3. In the case of napin, the thermal stability was kept all along the pH levels. Higher values of pH and temperature induced more unfolded structures through dividing the inter- and intra-chain disulfide bonds and promoting new molecular interactions. These new rearrangements led to a formation of a gels with a denser network and better mechanical properties comparable with those obtained from soy and legume protein [107]. He, et al. [108] observed that high-pressure treatments considerably raised the quantity of soluble protein aggregates of the rapeseed protein isolate, while heat treatments (80 and 100 °C), reduced them. The free sulfhydryl group content increased significantly after a 200 MPa pressure treatment, but 400 and 600 MPa treatments, as well as temperature treatments (60–100 °C), produced significant declines. This could be because disulfide bonds formed as the pressure-induced protein-protein interactions became more intense. The newly produced disulfide linkages promoted the protein aggregation, which helped the creation of high molecular weight proteins. The different ability of cruciferin and napin to form structures with diverse physicochemical features, that would promote a phase separation within the extruder fling doors, suggests its possible use as an ingredient for plant-based meat analogue development. Moreover, the high number of cysteine residues in napin, together with its low molecular weight and, hence, tiny size, make it an appealing candidate for replacing WG’s functionality. The rearrangements of disulfide bonds may occur in the extruder under extreme conditions (temperature and pressure), contributing to an increase in the elastic character of the composite generated from another plant source (pulses such as soy or pea) and rapeseed protein isolate. Recently, Jia, et al. [71] confirmed rapeseed protein concentrate as a favorable protein source for meat analogue production. The study revealed that rapeseed concentrate created fiber structures when processed above 140 °C through shear cell technology, and because of the high final fibrousness degree, it could also be used without WG. In baking, replacing part of the starch with rapeseed protein isolate (from 6 to 15%) caused an increase of instantaneous and viscoelastic compliance of the gluten-free dough system, while apparent viscosity dropped under applied shear [109]. Regarding the gluten-free breads, rapeseed protein incorporation helped to stabilize the gas bubbles during proofing, leading to samples with higher volume [110]. The same was reported by Salah, et al. [111] in gluten-free rice breads: rapeseed protein concentrate at a level above 6%, contributed to an improvement in the mass and alveolar structure compared to the control made with rice flour (100%). Given the encouraging results achieved with gluten-free baked goods, the surface activity and ability to stabilize the foam structure by rapeseed protein may be exploited in high-moisture shear processes to build a three-dimensional WG-like structure.

Among the global crops most employed for vegetable oil production, sunflower is surely in the top positions. After the oil extraction, the residue, also known as sunflower cake, can represent a valuable source of protein. Two major protein groups, 11S globulin (helianthinin) and 2S albumin (ratio of 2:1) compose the sunflower protein [112]. A particular relevance can be assumed by helianthinin, which is the major storage protein in sunflower seeds. It is composed of six subunits linked by twelve disulfide bonds, while 2S albumins are generally composed of a large and small polypeptide associated with two disulfide bonds [113]. With the objective of improving their functionality, Malik and Saini [114] applied heating near the isoelectric point (80 °C for 5, 15 and 25 min. at three pH values: 3.5, 4.5 and 5.5). The authors reported that around the isoelectric point (pH 4.5), the thermal stability of protein isolates was higher and was improved by the thermal treatment, while regarding the gel strength, those prepared with treated protein isolates were weaker than those obtained from the native protein isolates. Concerning the fiber formation, sunflower protein isolates were successfully electrospun in combination with polyvinyl alcohol (40:60) [115]. Jia, et al. [116] studied the structuring properties of de-oiled and pressed sunflower kernel after aqueous ethanol washing. Following this treatment, the resultant phenolic compounds content drastically reduced, even if it is not crucial for structuring processes. De-oiled sunflower kernels (40 wt.%) formed fibrous structures alone if processed via shear cell (140 °C), as opposed to pressed sunflower kernels that generated only a weak gel-like structure. According to the authors, this different structuring aptitude was due to the significant difference in the oil content, suggesting that proteinaceous raw materials with an oil content higher than 8% reduced the fiber formation.

Keeping with the oilseeds theme, seeds from hemp that contain sulfur-rich amino acids, can represent a potential alternative for the texturizing processes. In fact, globulin (edestin), which is the major storage protein in hemp seeds, consists of mostly 11S globulins (elevated levels of sulfur-containing amino acids), whereas the 2S albumin is formed by two polypeptide chains held together by two disulfide bonds [117]. To analyze the feasibility of hemp protein concentrate in replacing soy protein isolate for plant-based meat analogue production, Zahari, et al. [118] tested different substitution levels (20, 40 and 60%) at various moisture contents (65, 70 and 75%). Substitution levels up to 60% did not produce any significant change in terms of springiness, while samples obtained with 40% hemp protein concentrate and 70% moisture content showed the highest elasticity. In general, hemp protein concentrate absorbed less water and required higher temperatures to become denatured compared to soy protein isolate.

Nowadays, there is a growing interest in the use of pumpkin seed oil because of its high content of unsaturated fatty acids; owing to this, larger quantities of pumpkin seed cake (60–65% protein content) is being generated as a waste [119]. The major protein fraction of pumpkin seeds is represented by curcubitin (12S globulin), which consists of six subunits containing two disulfides connected by polypeptide chains. From the other side, albumin fractions include two small polypeptide chains connected via two disulfide links [120]. Regarding the functional properties of pumpkin seed protein isolate, its solubility reached the highest values at a pH around 8, while tensiometric examinations revealed that they can absorb at both air-water and oil-water interfaces under a wide pH range (from 3 to 8) [121]. Moreover, increasing the ionic strength caused protein solubility to decrease, but when the ionic strength was decreased, more stable emulsions were obtained due to more electrostatic repulsions between the oil droplets [122]. Such changes of functionalities observed at different conditions (pH and ion concentrations) may be of interest in a multi-phase system subjected to high-temperature and shear with a view to the formation of an anisotropic structure. Overall, the growing interest in oilseed protein for structuring processes is mainly due to their numbers of sulfur containing amino acids and relatively lower costs, being a side-stream of oil manufacturing. Further insights about the availability of free thiol groups for texturization, as well as the impact of antinutritional compounds that could interact with protein are needed to further improve the quality of the final products.

## 7. Conclusions

The manufacturing of plant-based meat analogues is not only about creating similar textures and mouthfeel to the whole animal muscle or processed meat products, but also about the integration of a complex set of properties, such as appearance, sensory attributes, nutritional quality, and food safety. To address these challenging requirements, an accurate examination of the ingredients and process is needed. Obtaining an anisotropic structure through high-shear wet processes, such as high-moisture extrusion or shear cell structuring, represents a critical factor for the development of high-quality meat analogues and is the result of the conformational changes on a molecular scale that take place during the manufacturing procedure. When submitted to thermomechanical processes, WG has been demonstrated to play a key role in the achievement of a meat-like structure. One of the main functionalities of WG is its higher propension in forming a viscoelastic phase on its own under high moisture and shearing conditions. Behind the formation of this three-dimensional network, disulfide bounds are the key pillars. Sulfur-rich amino acids, abundantly present in WG, are not only important for the coupling of two thiol groups within the WG chains, but they can also be reshuffled to form other covalent linkages within the other proteinaceous phase. WG’s action is also valuable during the fiber orientation stage, in which it controls the process by maintaining phase separation. Fiber formation is strongly dependent on process variables; for example, temperature increase during the process is related with a rise in polymerization degree, but at higher temperatures can lead to depolymerization. Water’s role as a plasticizer is crucial for the viscoelastic behavior of the melt and thermomechanical energy transfer, while screw speed and shearing rate help to define the fibrousness of final products. The knowledge of the physicochemical properties of the feedstocks and their impact on the processing variables represent a key aspect for meat alternative production. A better understanding of the chemical reactions produced during high-moisture thermomechanical treatments could generate added value to create plant-based meat analogues with characteristics increasingly closer to those of real meat ones. Given that the commercial meat alternatives principally include soy protein and WG in their formulations, future investigation should focus on finding and characterizing under-explored plant-based protein sources, potentially suitable for the development of a new generation of meat alternatives. From this perspective, zein offers novel characteristics that can be utilized in the creation of plant-based meat substitutes due to its propensity to induce phase separation and build elastic networks when subjected to heating conditions. Similar advantages have been reported about leaf proteins (RuBisCo) that can form firm gels thanks to their high sulfhydryl groups content. Oilseed proteins are potentially attractive due to their relative lower cost and structuring properties. In a multiphase system under high-moisture shear conditions, rapeseed protein may form its own phase with superior elastic and mechanical properties because of its higher water solubility and greater cysteine residues content. In conclusion, the ideal plant-based protein for gluten substitution in high-moisture shear processes should be rich in cysteine residues. Being capable of creating phase separation, having a highly elastic behavior, keeping the other phases apart during the alignment process, and promoting disulfide bonds with the other protein phase are pivotal aspects to consider for a gluten replacement within high-moisture shear processes. This could represent a valuable strategy for generating healthier products with a reduction in environmental stress and meat consumption.

## Figures and Tables

**Table 1:** Wheat gluten behavior under high-moisture shear conditions.

Process	Formulation	Process Variables	Main Results	Reference
Closed cavity rheometer	WG Moisture content: 20, 30, 40%	- Temperature: 30 to 170 °C. - Shear rate: 50 s^−1^, 25 s^−1^ and 0.1 s^−1^.	- Decreasing water content led to an onset increase in the reaction behavior temperature. - The influence of temperature on the rate of the reactions was a strong function of water content. - Samples with higher water content showed a significant increase in the rate of reactions.	Emin, et al. [42]
	WG Moisture content: 54%	- Temperature: 90, 100, 120, 140, 160 °C. - Process time: 18, 36, 60, 90, 180 min. - Shear rate: 0.1 s^−1^, 50 s^−1^.	- The molecular interactions (between sulfhydryl side chains and two cysteine residues) of WG were influenced by thermal and mechanical treatment. - In general, complex viscosity decreased when temperature increased. - Disulfide bond formation plays a key role in WG polymerization.	Pietsch, et al. [65]
	PPI, SPI and WG 30, 40, 50 and 60 wt.% Moisture content: >40%	- Temperatures: 30, 100, 120 and 140 °C. - Cooling: up to 30 °C. - Strain sweep: at constant frequency (1 Hz) and varied from 0.1 to 20 Hz. - Large amplitude oscillatory shear: strain amplitude 0.01–1000% at a constant frequency of 1 Hz.	- PPI and SPI at 40 wt.% behaved similarly, but different from WG. - The modulus of WG increased with increasing temperature and did not recover after cooling. - At 30 °C, PPI and SPI showed a higher dissipation ratio than WG. - Upon thermal treatment, PPI lost its elastic properties faster than SPI, while WG showed a rapid dissipation after extensive deformation.	Schreuders, et al. [66]
Shear cell	SPI-WG 20–40 wt.% 1:4, 2:3, 3:2, 4:1 Moisture content: >60%	- Shearing: 0 and 30 rpm. - Heating: 95 °C for 15 min. - Cooling: 4 °C for 30 min.	- SPI 30%: no anisotropy. No shearing: brittle and porous gel. Shearing: firm dense gel. - WG 30%: No shearing: randomly oriented small fibers. Shearing: evident fibrous structure. - SPI 15%–WG 15%: No shearing: gel with short thin fibers Shearing: gel with long, thick, and thin fibers in the shear direction. - SPI-WG (25 wt.%) samples with ratios 4:1, 3:2 were anisotropic. - SPI-WG (30 wt.%) samples with ratios 4:1, 3:2, 1:4 were anisotropic.	Grabowska, et al. [67]
	SPI-WG 3.3:1 Moisture content: 69%	- Temperature: 90–110 °C. - Process time: 5–25 min. - Rotation rate: 5–50 rpm.	- Samples treated at temperatures between 90 and 100 °C showed evident fibrousness, the role of process time and rotation rate is not critical under the described conditions.	Krintiras, et al. [68]
	SPI-WG 3.3:1 Moisture content: 69%	- Temperature: 120 °C. - Rotation rate: 15–45 rpm. - Process time: 10–50 min.	- Samples processed at 120 °C, 30 min and 20 rpm showed highly fibrous structures. - Samples treated at 10 rpm were not sufficiently structured, while at 50 rpm the samples were damaged and deformed. - Samples treated for 45 min showed burns.	Krintiras, et al. [15]
	SPI-WG 27.5, 30 and 35 wt.% 50:50 Moisture content: >60%	- Temperature: 95 °C. - Process time: 15 min. - Shearing: 30 rpm.	- SPI phase absorbed more water than WG phase. - Heating and/or shearing did not affect the water distribution in the SPI-WG blend.	Dekkers, et al. [69]
	SPI-WG 50:50 PPI-WG 50:50 Moisture content: 60%	- Temperature: 95 to 140 °C. - Shear rate: 39 s^−1^. - Process time: 15 min. - Cooling: 25 °C for 5 min.	- PPI-WG samples had a distinct fibrous morphology when sheared and heated at 120 °C. Processing at a lower temperature resulted in a weak product without fibers, while a higher temperature gave a strong and layered product. - SPI-WG samples obtained similar fibrous and anisotropic materials at a broader range of shearing temperatures (110–140 °C). - Mechanically, resultant blends with SPI were three times stronger than the blends with PPI at lower shearing temperatures (120 °C). At higher shearing temperatures (140 °C) the SPI-WG and PPI-WG products showed similar strength. - Decreased strength in SPI-WG and PPI-WG was observed in the samples treated at 130 °C. - Tensile strength of PPI-WG (110 to 130 °C) samples was the closest to that of chicken meat.	Schreuders, et al. [70]
	RPC-WG 20:20 and WG up to 32% Moisture content: 60%	- Temperature: 85 to 150 °C. - Shearing: 30 rpm. - Process time: 15 min. - Cooling: 25 °C for 15 min.	- At 150 °C, a gel-like structure with tiny fibers was formed, while at 95 and 120 °C, the gels showed a crumble structure. - Increased WG resulted in more noticeable fibrousness with microscopic fibers, even more so than WG-only products. - The addition of WG to SPC increased the anisotropy, tensile stress and strain.	Jia, et al. [71]
High-moisture extrusion	WG only Moisture content: 40%	- Screw speed: 300 rpm. - Feed rate of 10 kg/h. - Barrel temperatures 1–7 from 40 to 170 °C. - Cooling temperature: 0, 20, 50, 80 °C.	- Only temperatures from 90 to 160 °C influenced WG polymerization, while die temperature, pressure and specific mechanical energy had no significant influence.	Pietsch, et al. [24]
	WG only Moisture content: 54%	- Screw speeds: 180, 400 and 800 rpm. - Feed rates: 10 and 20 kg/h. - Barrel temperatures: 1–7 from 40 to 165 °C. - Cooling die: 50 °C.	- WG polymerization increased with the increasing thermomechanical treatment. - The presence of a dispersed phase contributed to the formation of anisotropic product structures. - Increase in WG polymerization could be correlated with the formation of anisotropic product structures and an increase in hardness and Young’s modulus.	Pietsch, et al. [72]
	SPC-WG (10, 20, 30%) Moisture content: 60%	- Barrel temperatures: 20, 50, 80, 110, 150, 170, 50 °C + cooling die. - Screw speed: 400 rpm. - Feed rate: 2.8 kg/h.	- Samples containing 30% of WG showed the highest degree of texturization, fibrous structure, hardness, and chewiness. - Samples containing 20 and 30% of WG exhibited a microstructure with large fibrous structures. - Disulfide bonds increased as the quantity of WG increased.	Chiang, et al. [73]
	SPI-WG-Corn Starch 90:0:10 50:40:10 Moisture content: 30 and 70%	- Temperatures: 100, 160 and 130 °C. - Screw speeds: 150 and 200 rpm. - Feed rate: 100 g/min.	- Extrusion type and WG incorporation had a major impact on the physicochemical properties of the samples. - Screw speed affected only the springiness of the high-moisture wet extrudates. - Low-moisture extrusion cooking without addition of WG produced an expanded protein structure with the lowest texture stability after cooking. - WG incorporation under low-moisture conditions produced samples with a spongy structure. - Samples at high-moisture conditions with WG generated samples with a denser structure with more fibers and texture stability.	Samard, et al. [74]
	SPI-MBPI-PNPI - PPI-WG Moisture content: 50%	- Temperature: 100, 160 and 140 °C. - Screw speed: 250 rpm. - Feed rate: 100 g/min.	- PPI-based samples formed a sponge-like structure that had desirable rehydration and textural properties with good oil absorption and emulsion properties. - SPI and WG-based samples showed better textural properties. - MBPI and PNPI-based samples were lower in rehydration and textural properties. - PPI-based TVP showed the highest quality.	Samard, et al. [75]
	- SP-WG (10, 15, 20, 25, 30%) Moisture content: 40 to 60%	- Temperature: 130, 140, 150, 160 and 170 °C. - Feed rate: 6 kg/h.	- Moisture content increase from 40 to 55% led to elasticity decrease. - Temperature increase from 130 to 160 °C increased the creep deformation of the extrudate. - The addition of WG improved the organized structure of the samples. - Microstructure and morphology of the samples were affected by moisture content, extrusion temperature and WG content. - The fractal dimension of the samples decreased with an increase in moisture content and WG content reflecting a more aligned structure.	Wu, et al. [25]
	SPI-WG (10, 20, 30, 40%) Moisture content: 50 to 80%	- Temperature: 20, 50, 80, 150, 140, 100, 80 and 60 °C. - Feed rate: 30 g/min.	- Samples with higher WG content and lower moisture content retained more volatile flavor and water. - The structure of the samples with higher WG and moisture content had a tight structure with no breakage phenomenon (cracks in the outer structure).	Guo, et al. [76]
	WG Moisture content: 70%	- Temperature: 80–140 °C. - Screw speed: 40 to 80 rpm. - Feed rate: 30 to 50 g/min.	- Screw speed and flow rate increase corresponded to a higher formation of high molecular weight glutenin subunits while free sulfhydryl groups and low molecular weight glutenin subunits decreased. - At higher extrusion temperatures a homogeneous and denser WG network was formed.	Jia, et al. [77]
	SPC-WG (30, 50, 70, 100%) Moisture content: 60%	- Temperature: 40, 60, 80, 100, 120, 150 and 150 °C. - Screw speed: 150 rpm - Feed rate: 8 g/min	- Fibrous degree increased with WG addition (until 50%). - α-helix content decreased with increasing WG content. - β-sheet content increased with increasing WG content. - Hydrogen bonding increased with the increasing WG addition. - SPC-WG ratio of 50/50 resulted in the best fibrous structure.	Zhang, et al. [78]

## Data Availability

Not applicable.
